# Clinical implications of Delphian lymph node metastasis in papillary thyroid carcinoma: a single-institution study, systemic review and meta-analysis

**DOI:** 10.1186/s40463-019-0362-7

**Published:** 2019-08-30

**Authors:** Bin Wang, Xing-zhu Wen, Wei Zhang, Ming Qiu

**Affiliations:** Department of General Surgery, Changzheng Hospital, Second Military Medical University, No. 415 in Fengyang Road, Huangpu District, Shanghai, 200003 China

**Keywords:** Delphian lymph node, Papillary thyroid carcinoma, Clinical characteristics

## Abstract

**Background:**

To evaluate the possible predictive value and clinicopathological characteristics of Delphian lymph node metastasis in papillary thyroid carcinoma.

**Methods:**

A retrospective analysis of papillary thyroid carcinoma patients with Delphian lymph node metastasis in a single institution and meta-analysis of literature reports were performed.

**Results:**

In own series, Delphian lymph node metastasis was detected in 19 (9.9%) of 192 papillary thyroid carcinoma patients and was significantly associated with tumor size≥1 cm (*P* = 0.003), multifocality (*P* = 0.006) and extrathyroid extension (*P* < 0.001) in the multivariate analysis. Female was a protective factor for Delphian lymph node metastasis (*P* = 0.001). Delphian lymph node metastasis was highly predictive of further central lymph node metastasis (positive predictive value = 89.5%, negative predictive value = 67.6%) and moderately predictive of lateral lymph node metastasis (positive predictive value = 26.3%, negative predictive value = 95.4%). In this meta-analysis, there was a strong correlation between Delphian lymph node metastasis and aggressive clinicopathologic characteristics with regards to multifocality (*P* = 0.0008), bilaterality (*P* = 0.04), extrathyroid extension (*P* < 0.00001), lymphovascular invasion (*P* < 0.00001), further central lymph node metastasis (*P* < 0.00001) and lateral lymph node metastasis (*P* < 0.00001).

**Conclusions:**

This single-institution observational study and meta-analysis identified that Delphian lymph node metastasis was significantly associated with unfavorable clinicopathological characteristics and had a strong predictive power for further disease in the central compartment.

**Trial registration:**

The clinical study was retrospectively registered to UMIN clinical trials registry (the registry number: UMIN000033835).

**Electronic supplementary material:**

The online version of this article (10.1186/s40463-019-0362-7) contains supplementary material, which is available to authorized users.

## Introduction

The Delphian lymph node (DLN), also called the prelaryngeal or cricothyroid node, locates in the fascia above the thyroid isthmus and lies between the cricoid and thyroid cartilages [1, 2]. The DLN receives afferent lymph flow from the larynx and the thyroid gland, then flows towards the central and lateral lymph nodes [2]. Therefore, DLN status should be critically evaluated in patients with cancers involving the larynx, hypopharynx and thyroid. Most studies to date on the DLN address its significance in laryngeal and hypopharyngeal carcinoma and emerging evidence suggests that the positive DLN is a poor prognostic factor in laryngeal and hypopharyngeal cancers [1, 3–9]. Unlike laryngeal cancer, the clinicopathologic factors of the positive DLN in thyroid cancer are still not completely understood. To our knowledge, there are 9 reports addressing the predictive value of DLN metastasis in papillary thyroid carcinoma (PTC) [10–18] and most of these studies were published in recent years. The clinical data from some studies indicate that DLN metastasis is a predictor of further disease in the central compartment and in the lateral compartment. However, some investigators contended that the DLN is frequently found without extensive lymph node disease and even argued that DLN involvement is a misleading and unreliable sign [19, 20].

Because of inconsistent results and lack of meta-analyses to systematically review the significance of the DLN in PTC, we report the outcomes of a single-institution case series and present the first meta-analysis of the clinical characteristics of PTC patients with DLN metastasis.

## Materials and methods

### Single-institution observational study

We retrospectively reviewed the medical records of 192 patients with a final diagnosis of PTC who underwent total thyroidectomy and central lymph node dissection (CLND) with or without lateral lymph node dissection (LLND) in Shanghai Changzheng Hospital (Shanghai, China) between July 2017 and August 2018. The protocol for this research project was approved by the local Clinical Ethics Committee and written informed consent was obtained from each participant. The clinical study was registered to UMIN clinical trials registry (the registry number: UMIN000033835) (Additional file 3).

CLND was performed routinely on the affected side. CLND on the contralateral side was performed when any of the central lymph nodes were found to be suspicious on preoperative imaging examination or upon intraoperative inspection. LLND was performed only if preoperative fine needle aspiration cytology confirmed evidence of metastasis. The soft tissue and lymph nodes between the thyroid and cricoid cartilage were excised in all 192 cases, then were routinely labeled as DLNs and examined by frozen section evaluation and separate histopathological examinations. Most of the frozen sections were interpreted by three subspecialty pathologists who all had rich experiences with the pathological diagnosis of PTC and lymph nodes.

The relationship between DLN metastasis and gender, age, tumor size, multifocality, thyroiditis, BRAF^V600E^ mutation, extrathyroid extension (ETE), tumor location, serum thyroglobulin (Tg) level, thyroglobulin antibody (TgAb) level, thyroid peroxidase (TPO) antibody level, thyroid stimulating hormone (TSH) level, central lymph node metastasis (CLNM), lateral lymph node metastasis (LLNM) was gathered.

### Systematic review and meta-analysis

Electronic searches were performed for relevant reports in two databases (PubMed and Embase) with publication date before August 2018. The search strategy used the following terms: (Delphian lymph node OR prelaryngeal lymph node OR cricothyroid lymph node) AND (thyroid tumor OR thyroid cancer OR thyroid carcinoma OR thyroid neoplasm). The abstracts of all potential articles, references, and related articles were reviewed according to their titles and each article was independently assessed for eligibility using inclusion criteria.

Inclusion criteria were as follows: (1) comparative studies of the clinicopathologic parameters of the positive DLN and the negative DLN in PTC; (2) articles published in English before August 2018 and (3) exact and intact dichotomous-type or continuous-type data with standard deviations. The literature search was independently performed by 2 authors. The third author determined whether to include the study when controversy occurred.

Two investigators independently extracted and collected data using a standardized data-extraction protocol. For repeated publications, the latest data about interesting outcomes were extracted. For example, Zheng et al. reported their results twice at different periods of the same trial [18, 21]. Two authors (BinWang and Xing-zhu Wen) independently assessed the quality of the included studies according to the Newcastle–Ottawa Scale (NOS) [22]. Disagreements regarding methodological assessment were discussed and resolved through consensus.

### Statistical analysis

For our single-institution experience, the database was exported to SPSS software (version 19.0; IBM-SPSS, Inc., Chicago, IL, USA). Continuous data was compared using Student t-test and categorical variables were compared using Pearson’s chi-squared or Fisher’s exact test. Variables with *p* < 0.15 in univariate analysis were considered statistically significant and included in the multivariate analysis. Differences were considered significant when *P* < 0.05.

Regarding meta-analysis, data extracted from the included trials were integrated with Review Manager Software version 5.3 (Cochrane Collaborative, Oxford, UK). The odds ratio (OR) was used for dichotomous-type data. The ORs were combined using a random-effects model. Heterogeneity among articles was quantitatively assessed using the Q test and I^2^ statistic [23]. A significant heterogeneity was defined as I^2^ > 50% or Q-test reporting a *P* value < 0.05. Sensitivity analysis was applied by removing individual studies from the data set and evaluating the effect of their removal on the pooled OR. Publication bias was examined by Begg’s funnel plot as well as Egger’s linear regression test.

## Results

### Single-institution experience

All patients underwent CLND and 13 patients underwent LLND. DLN metastasis was observed in 19 patients (9.9%) and only 1 patient (0.5%) had DLN metastasis without other compartments metastasis. A total of 101 lymph nodes among the patients with DLN metastasis and 799 lymph nodes among the patients without DLN metastasis were detected. No significant difference existed between the 2 groups regarding the mean number of detected lymph nodes (*P* = 0.320). Histopathological examination proved 65 metastatic lymph nodes among the patients with DLN metastasis and 145 metastatic lymph nodes among the patients without DLN involvement. The mean number of metastatic lymph nodes among the patients with DLN metastasis was significantly higher than that among patients without DLN involvement (*P* < 0.001). In the univariate analysis, DLN metastasis was significantly associated with tumor size≥1 cm (*P* < 0.001), multifocality (*P* = 0.002), ETE (P < 0.001), CLNM (*P* < 0.001) and LLNM (*P* = 0.004; Table 1), while there was no significant correlation between DLN metastasis and the following variables: tumor distribution (*P* = 0.152), tumor location (*P* = 0.443), bilaterality (*P* = 0.061), BRAF^V600E^ mutation (*P* = 0.383), Hashimoto’s thyroiditis (*P* = 0.098), and preoperative TSH level (*P* = 0.590; Table 1). The proportion of female patients (*P* = 0.007) and patients aged ≥45 years (*P* = 0.042), was lower in PTC patients with the positive DLN than in those with the negative DLN (Table 1).

**Table 1:** Comparison of clinicopathological characteristics between patients with or without DLN metastasis

Characteristics	DLN-positive (*n* = 19)	DLN-negative (*n* = 173)	*P* value
Female %(n)	42.1 (8)	72.3 (125)	0.007
Age ≥ 45 years %(n)	31.6 (6)	56.1 (97)	0.042
Tumor size ≥ 1 cm %(n)	73.7 (14)	28.3 (49)	< 0.001
Tumor distribution			0.152
left side %(n)	21.1 (4)	32.4 (56)	
right side %(n)	42.1 (8)	50.3 (87)	
double side %(n)	36.8 (7)	17.3 (30)	
Tumor location			0.443
isthmus/upper third %(n)	31.6 (6)	28.9 (50)	
middle third %(n)	26.3 (5)	40.5 (70)	
lower third %(n)	42.1 (8)	30.6 (53)	
Multifocality %(n)	68.4 (13)	32.9 (57)	0.002
Bilaterality %(n)	36.8 (7)	17.3 (30)	0.061
ETE %(n)	42.1 (8)	5.2 (9)	< 0.001
BRAF^V600E^ mutation %(n)	31.6 (6)	21.4 (37)	0.383
Hashimoto’s thyroiditis %(n)	52.6 (10)	33.5 (58)	0.098
Tg level (ng/ml)	24.32 ± 28.70	24.22 ± 35.22	0.991
Tg-Ab level (IU/ml)	95.56 ± 164.79	83.66 ± 167.50	0.769
TPO level (IU/L)	45.29 ± 58.74	43.53 ± 93.32	0.936
TSH level (mIU/L)	2.69 ± 1.50	2.90 ± 1.65	0.590
CLNM %(n)	89.4 (17)	32.4 (56)	< 0.001
Lymph nodes dissected (n)	5.32 ± 2.34	4.62 ± 2.94	0.320
Metastatic lymph nodes (n)	3.42 ± 1.84	0.84 ± 1.78	< 0.001
LLNM %(n)	26.3 (5)	4.6 (8)	0.004
Mean no. of LLNM (n)	1.16 ± 2.34	0.21 ± 1.11	0.099

*DLN* Delphian lymph node*, ETE* Extrathyroid extension, *Tg* Thyroglobulin, *Tg-Ab* Thyroglobulin antibody, *TPO* Thyroid peroxidase, *TSH* Thyroid stimulating hormone, *CLNM* Central lymph node metastasis, *LLNM* Lateral lymph node metastasis

In univariate analysis, female, age ≥ 45 years, tumor size, multifocality, bilaterality, ETE and Hashimoto’s thyroiditis showed a *p* < 0.15. These factors thus were included in the multivariate analysis (Table 2). The multivariate analysis revealed that tumor size≥1 cm (*P* = 0.003), multifocality (*P* = 0.006) and ETE (*P* < 0.001) were found to be independent risk factors of DLN metastasis. Female was a protective factor for DLN metastasis (*P* = 0.001). For further CLNM and LLNM, DLN status had sensitivity, specificity, positive and negative predictive values of 23.3, 98.3, 89.5 and 67.6, 38.5, 92.2, 26.3, and 95.4%, respectively (Table 3). Compared with patients without DLN metastasis, patients with DLN metastasis were approximately 13.7 times more likely to have further CLNM and 4.9 times more likely to have LLNM.

**Table 2:** Multivariate logistic regression analysis for DLN metastasis

Variables	Sig.	Exp (B)	95.0% CI Exp (B)	
			Lower	Upper
Age ≥ 45 years	0.137	0.359	0.093	1.384
Female	0.001	0.079	0.018	0.343
Tumor size ≥ 1 cm	0.003	8.255	2.023	33.680
multifocality	0.006	8.034	1.803	35.796
bilaterality	0.657	0.706	0.152	3.275
ETE	< 0.001	15.772	3.500	71.067
Hashimoto’s thyroiditis	0.158	2.445	0.707	8.447

*ETE* Extrathyroid extension

**Table 3:** Ability of DLN metastasis to predict further CLNM and LLNM

LNM types	Sensitivity(%)	Specificity(%)	PPV(%)	NPV(%)	LR+	LR-
Central	23.3	98.3	89.5	67.6	13.7	0.8
Lateral	38.5	92.2	26.3	95.4	4.9	0.7

*LNM* Lymph node metastasis, *PPV* Positive predictive value, *NPV* Negative predictive value, *LR* Likelihood ratio

### Systematic review and meta-analysis

**Fig. 1:**
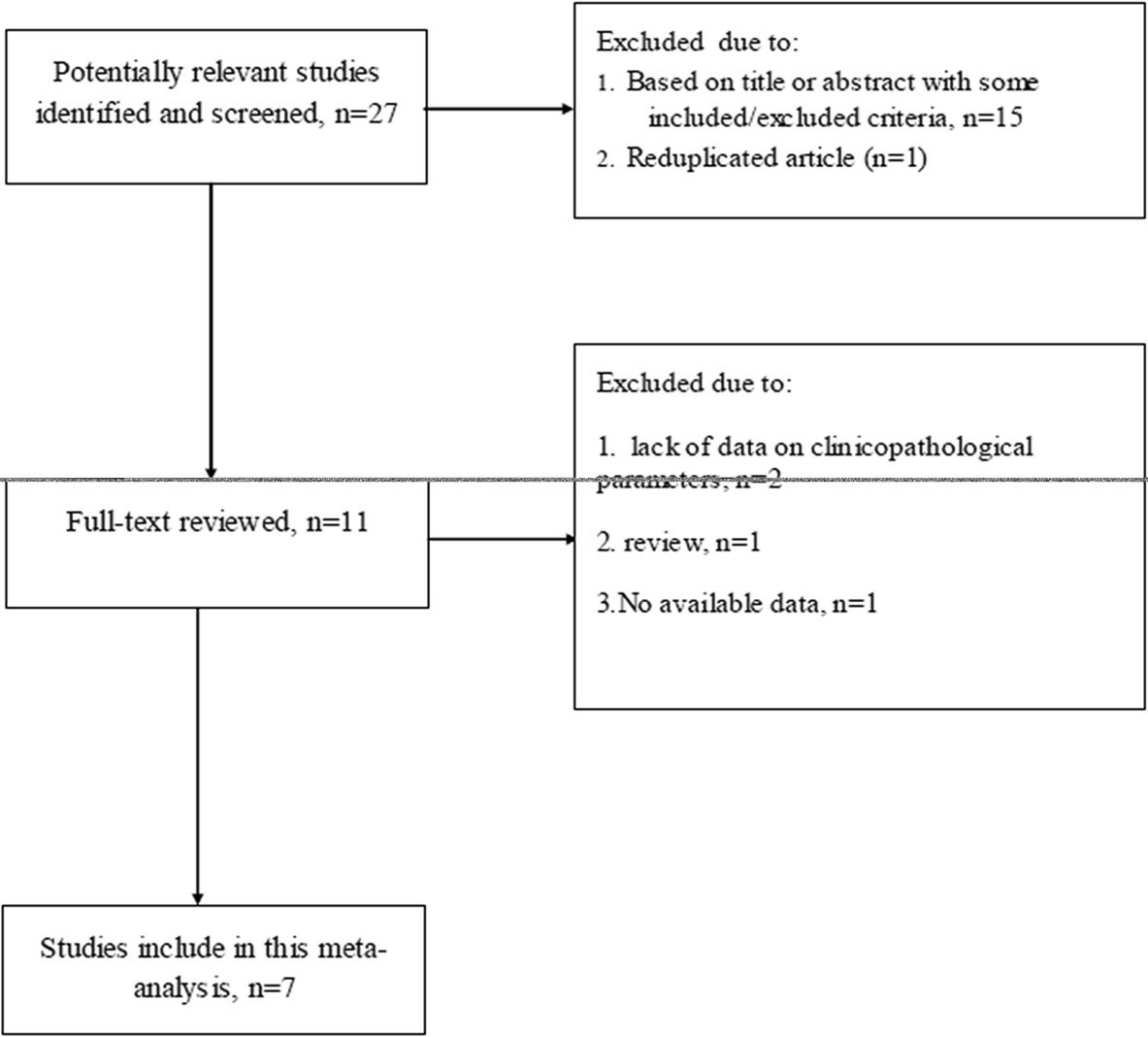
Flow diagram of article selection for this meta-analysis

The procedure used for study screening and selection was shown in Fig. 1. Finally, the literature search identified 7 studies [12–18] published between May 2011 and February 2017 for this meta-analysis. The eligible studies were summarized in Table 4. Among the PTC patients, 247 (16.2%) were found to have DLN metastasis. The quality scores of the 7 studies ranged from 5 to 7 with a mean of 6.0.

**Table 4:** Characteristics of individual studies included in the meta-analysis

Reference	Country	Sample capcity	Incidence of DLN metastasis(%)	Quality score
Chai 2014 [12]	Korea	46/324	12.4	7
Lyer 2011 [13]	USA	25/76	24.8	6
Kim 2012 [14]	Korea	53/255	17.2	5
Lee 2013 [15]	Korea	13/54	19.4	6
Oh 2013 [16]	Korea	49/196	20.0	6
Tan 2017 [17]	China	19/212	8.2	6
Zheng 2017 [18]	China	42/164	20.4	6

*DLN* Delphian lymph node

**Fig. 2:**
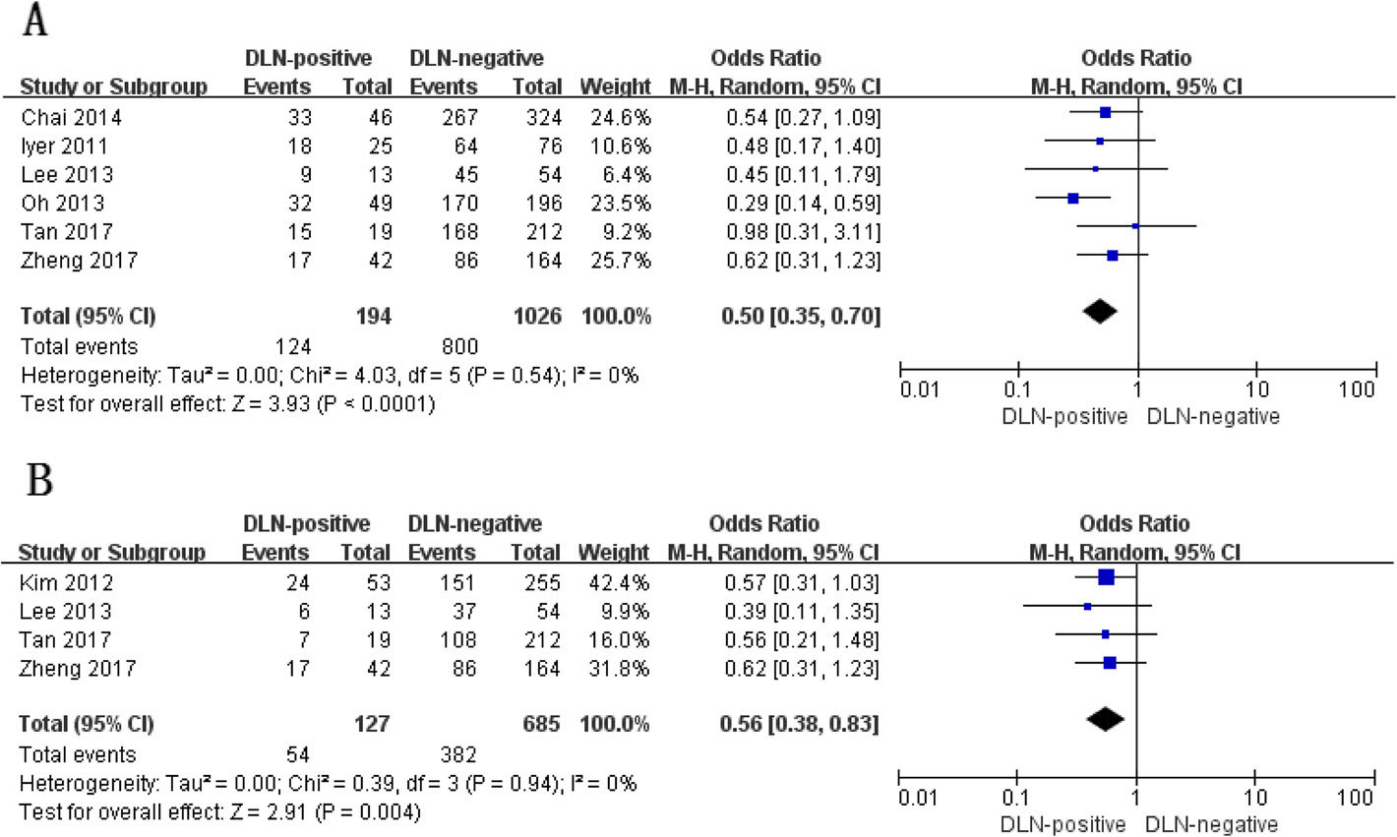
Demographic characteristics with regard to age (**a**) and gender (**b**) of papillary thyroid carcinoma patients with and wit7hout Delphian lymph node metastasis

In total, 6 studies were comparable in terms of gender [12, 13, 15–18] and 4 studies reported the prevalence of patients aged ≥45 years [14, 15, 17, 18]. The proportion of female and patients aged ≥45 years was significantly lower in PTC patients with the positive DLN than in those with the negative DLN (female: OR, 0.50; *P* < 0.001; Fig. 2a; aged ≥45 years: OR, 0.56; *P* = 0.004; Fig. 2b). There was no statistically significant heterogeneity in both analyses (female: P for heterogeneity =0.54, I^2^ = 0%, Fig. 2a; aged ≥45 years: P for heterogeneity =0.94, I^2^ = 0%, Fig. 2b).

**Fig. 3:**
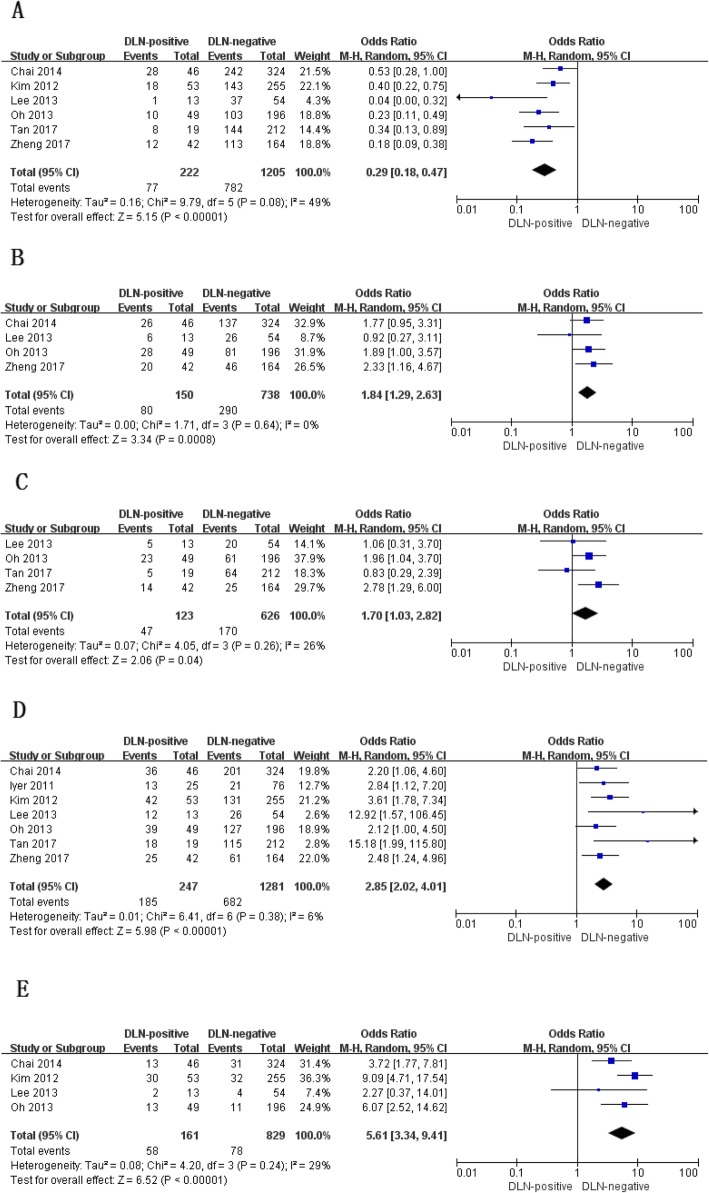
Clinicopathologic characteristics with regard to papillary thyroid microcarcinoma (**a**), multifocality (**b**), bilaterality (**c**), extrathyroid extension (**d**) and lymphovascular invasion (**e**) of papillary thyroid carcinoma tumor with and without Delphian lymph node metastasis

The prevalence of PTMC was analyzed in 6 studies [12, 14–18]. The prevalence of PTMC was 34.7% (77/222) in patients with DLN metastasis and 64.9% (782/1205) in those without DLN involvement. The difference was statistically significance (OR, 0.29; *P* < 0.00001; Fig. 3a). There was no statistically significant heterogeneity among the studies (P for heterogeneity =0.08, I^2^ = 49%, Fig. 3a).

Four studies reported the percentages of multifocality [12, 15, 16, 18] and bilaterality [15–18]. Tumor multifocality and bilaterality were more common in patients with DLN metastasis than in those without DLN involvement (multifocality: OR, 1.84; *P* = 0.0008; Fig. 3b; bilaterality: OR, 1.70; *P* = 0.04; Fig. 3c). Statistically significant heterogeneity failed to be detected in both analyses (multifocality: P for heterogeneity = 0.64, I^2^ = 0%; Fig. 3b; bilaterality: P for heterogeneity = 0.26, I^2^ = 26%; Fig. 3c).

All of 7 studies included [12–18] reported the prevalence of patients with ETE and 4 studies including addressed the frequency of lymphovascular invasion [12, 14–16]. ETE and lymphovascular invasion were both more prevalent in DLN-positive patients (ETE: OR, 2.85; *P* < 0.00001; Fig. 3d; lymphovascular invasion: OR, 5.61; P < 0.00001; Fig. 3e). No statistically significant heterogeneity existed in both analyses (ETE: P for heterogeneity =0.38, I^2^ = 6%, Fig. 3d; lymphovascular invasion: P for heterogeneity =0.24, I^2^ = 29%, Fig. 3e).

**Fig. 4:**
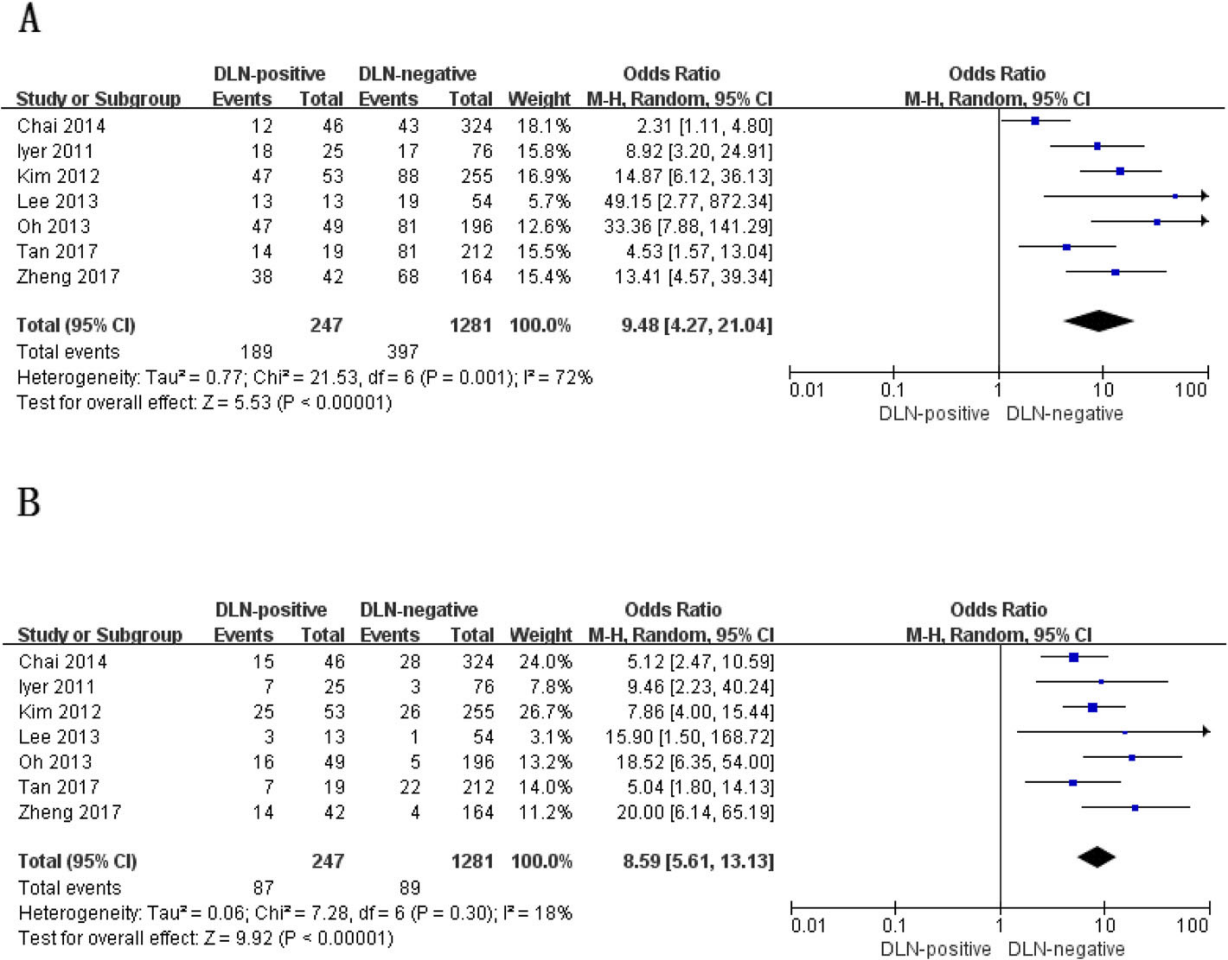
Other compartment metastasis with regard to central (**a**) and lateral (**b**) lymph node metastasis of patients with papillary thyroid carcinoma with and without Delphian lymph node metastasis

All of the 7 studies included evaluated the percentages of CLNM and LLNM [12–18], and the pooled data showed that CLNM and LLNM both occurred more commonly in PTC patients with DLN metastasis than those without DLN involvement (CLNM: OR, 9.48; *P* < 0.00001; Fig. 4a; LLNM: OR, 8.59; *P* < 0.00001; Fig. 4b). Statistically significant heterogeneity was detected in the meta-analysis of CLNM (P for heterogeneity =0.001, I^2^ = 72%, Fig. 4a) whereas no statistically significant heterogeneity was present in the meta-analysis of LLNM (P for heterogeneity =0.30, I^2^ = 18%, Fig. 4b).

**Fig. 5:**
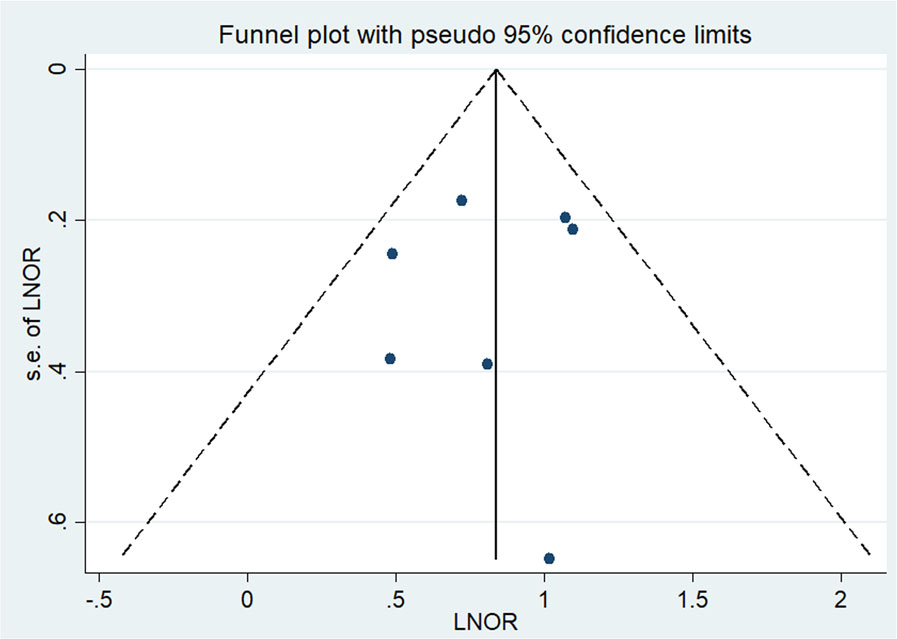
Funnel plot of standard error by log odds ratio

The sensitivity analyses revealed that the study of Zheng et al. [18] influenced the bilaterality result. After exclusion of this study, the prevalence of bilaterality failed to show significant difference between DLN-positive PTCs and DLN-negative PTCs (OR, 1.43; *P* = 0.195; see Additional file 1: Figure S1). The study of Chai et al. [12] influenced the heterogeneity of further CLNM. After exclusion of this study, the significant heterogeneity vanished statistically (P for heterogeneity =0.22, I^2^ = 29%) and no change in the result with regard to CLNM (OR, 12.02; 95% CI, 6.78–21.32, P < 0.00001; see Additional file 2: Figure S2) was observed. Other studies did not affect the pooled ORs. In the funnel plots and the Egger’s regression tests, there was no evidence of publication bias (Fig. 5).

## Discussion

This single-institution observational study and meta-analysis showed that DLN metastasis was less common in women with PTC but had a positive association with aggressive clinicopathological characteristics of PTC such as larger primary tumor, ETE, multifocality, and other compartment metastasis.

The name “Delphian” originated from a priestess in Delphi who can foresee the future and was first used to name the lymph node by Raymond B. Randall, a senior student of Harvard Medical School, in 1948 [1, 17]. As its name indicates, the DLN can predict the progression of thyroid cancer. The data in this single-institution showed patients with DLN metastasis was approximately 13.7 times more likely to have further CLNM and 4.9 times more likely to have LLNM. A study of Isaacs et al. [11] indicated that DLN positivity predicted a 9-fold higher frequency of LLNM, and a 40-fold higher frequency of any nodal disease.

This meta-analysis revealed a strong correlation between DLN metastasis and aggressive clinicopathologic characteristics such as ETE, multifocality, lymphovascular invasion, and further central and lateral compartment metastasis. In own series, tumor size≥1 cm, multifocality and ETE were found to be independent risk factors of DLN metastasis. Previous studies also investigated the risk factors for DLN metastasis but the conclusions were inconsistent. Oh et al. [16] revealed that lymphovascular invasion and tumor size played key roles in the occurrence of DLN metastasis. Chai et al. [12] found that tumor location in the isthmus or upper third of the thyroid was a predictor for DLN metastasis. Tan et al. [17] identified capsular invasion as an independent risk factor for DLN metastasis by multivariate analysis. Zheng et al. [18] reported that BRAF mutation was correlated with DLN metastasis. Kim et al. [14] demonstrated thyroiditis contributes to inhibiting DLN metastasis. This single-institution study showed that female played a role in impeding DLN metastasis. The inconsistent results may be attributed to the differences in sample size or demographics of patients included in each study.

In laryngeal cancer, the presence of DLN metastasis increases LLNM, resulting in a high recurrence rate and low survival rate [1, 24]. In PTC with DLN metastases, lymph node metastasis are detected in up to 95.9% of the central lymph nodes [16] and 47.2% of the lateral lymph nodes [14]. According to nodal staging for thyroid cancer, N1a refers to metastatic disease in the central compartment and N1b refers to metastasis to the lateral nodal chains. Because DLN positivity is predictive of lateral compartment disease, Delbridge et al. have suggested that nodal metastasis to the DLN should be upstaged to N1b [10]. DLN status has important implications in extending the scope of surgical procedures, planning radiotherapy, and determining outcome. Current methods for evaluating preoperative DLN status such as ultrasonography, computed tomography, or magnetic resonance imaging are imperfect [12, 25] because the median size of the positive DLN is small [13]. Intraoperative frozen section is generally accepted as one of the sensitive and useful tools for evaluation of nodal status of DLN. The current American Thyroid Association guidelines [26] do not recommend prophylactic CLND for small tumors (T1 or T2 classification). However, even in cases of PTC with small tumors, there was a high rate of CLNM [27]. Therefore, DLN could be sent for frozen section evaluation because of its predictive value for widespread nodal metastasis [13]. If the DLN is positive, CLND should be carefully considered even in clinically node-negative PTC.

There are few reports addressing the recurrence of PTC with DLN metastasis [12, 16, 18]. Studies showed that PTC recurrence was slightly higher in DLN-positive than in DLN-negative patients, although the difference did not reach statistical significance [12, 16]. However, Zheng et al. [18] showed that DLN-positive patients had a significantly higher rate of unstimulated Tg ≥1 ng/ml than DLN-negative patients during a median follow-up duration of 14 months and 11 months for DLN-positive patients and DLN-negative patients, respectively. Metastasis to the DLN is a poor prognostic factor in many malignant neck cancers [24, 25], and it is associated with several poor prognostic factors in PTC, including ETE [28], and a heavier nodal burden, in terms of number of metastatic nodes and node size [29]. These factors could act as confounders for the relationship between DLN metastasis and PTC prognosis [30]. To date, there is no published evidence or definitive studies on the association between survival and DLN involvement in PTC. Owing to the limited data on long-term follow-up of DLN metastasis in PTC, its relationship with the recurrence and survival of PTC patients remains unclear. Therefore, further studies with longer follow-up periods are warranted to explore the prognostic significance of DLN metastasis in PTC.

The present study had several limitations. First, this study was a 1 year retrospective study in a single institution. Some new findings in this study such as female as a protective factor for DLN metastasis might be not completely convincing and needed more perspective studies with large sample size and relatively long-term follow-up periods to confirm. Second, the sample size in the meta-analysis and the single-institution observational study was not well-matched because the rate of DLN metastasis was low. In this study, the ratio of DLN positive and negative patients was 19/173, which resulted to the low power of test statistic. Third, most studies were performed in Korea and China. Therefore, the results may not accurately reflect the clinical characteristics of PTC in another region.

## Conclusion

In conclusion, the results of this single-institution observational study and meta-analysis showed that the PTCs with DLN metastasis were characterized by larger primary tumor, multifocality, bilaterality, ETE, lymphovascular invasion, and other compartment lymph node metastasis. The data of this single-institution study identified that tumor size≥1 cm, multifocality and ETE were independent risk factors of DLN metastasis and DLN metastasis was highly predictive of further CLNM and moderately predictive of LLNM. The intraoperative frozen section of DLN in PTC patients could be considered based on the risk factors above mentioned. Future prospective multi-institutional studies are needed to determine whether prophylactic CLND is essential If DLN is confirmed to be positive during surgery.

## Additional files

Additional file 1: **Figure S1.** The sensitivity analysis of bilaterality with and without Delphian lymph node metastasis. (PNG 771 kb)

Additional file 2: **Figure S2.** The sensitivity analysis of further central lymph node metastasis with and without Delphian lymph node metastasis. (PNG 836 kb)

Additional file 3: Ethics approval from the local Clinical Ethics Committee. (JPG 59 kb)

## Data Availability

The datasets used and/or analyzed during the current study are available from the corresponding author on reasonable request.

## Abbreviations

CLND: Central lymph node dissection
CLNM: Central lymph node metastasis
DLN: Delphian lymph node
ETE: Extrathyroid extension
LLND: Lateral lymph node dissection
LLNM: Lateral lymph node metastasis
NOS: Newcastle–Ottawa Scale
OR: Odds ratio
PTC: Papillary thyroid carcinoma
Tg: Thyroglobulin
TgAb: Thyroglobulin antibody
TPO: Thyroid peroxidase

## References

[1] Olsen KD, DeSanto LW, Pearson BW (1987). Positive Delphian lymph node: clinical significance in laryngeal cancer. Laryngoscope..

[2] Nakayama M, Seino Y, Okamoto M, Mikami T, Okamoto T, Miyamoto S (2011). Clinical significance of positive Delphian node in supracricoid laryngectomy with cricohyoidoepiglottopexy. Jpn J Clin Oncol.

[3] Thaler ER, Montone K, Tucker J, Weinstein GS (1997). Delphian lymph node in laryngeal carcinoma: a whole organ study. Laryngoscope..

[4] Resta L, Micheau C, Cimmino A (1985). Prognostic value of the prelaryngeal node in laryngeal and hypopharyngeal carcinoma. Tumori..

[5] Foote RL, Buskirk SJ, Stanley RJ, Grambsch PM, Olsen KD, DeSanto LW (1989). Patterns of failure after total laryngectomy for glottic carcinoma. Cancer..

[6] Tomik J, Skladzien J, Modrzejewski M (2001). Evaluation of cervical lymph node metastasis of 1400 patients with cancer of the larynx. Auris Nasus Larynx.

[7] Pignataro L, Peri A, Pagani D, Scaramellini G, Broich G (2003). Prognostic value of Delphian lymph node in T1b glottic carcinoma. Anticancer Res.

[8] Kocatürk S, Okuyucu S, Köybaşioğlu F, Kaçar A, Erkam U (2003). The importance of the Delphian lymph node in subtotal laryngeal surgery. Kulak Burun Bogaz Ihtis Derg.

[9] Luna-Ortiz K, Pasche P, Tamez-Velarde M, Villavicencio-Valencia V (2009). Supracricoid partial laryngectomy with cricohyoidoepiglottopexy in patients with radiation therapy failure. World J Surg Oncol.

[10] Isaacs JD, Lundgren CI, Sidhu SB, Sywak MS, Edhouse PJ, Delbridge LW (2008). The Delphian lymph node in thyroid cancer. Ann Surg.

[11] Isaacs JD, McMullen TP, Sidhu SB, Sywak MS, Robinson BG, Delbridge LW (2010). Predictive value of the Delphian and level VI nodes in papillary thyroid cancer. ANZ J Surg.

[12] Chai YJ, Kim SJ, Choi JY, Koo do H, Lee KE, Youn YK (2014). Papillary thyroid carcinoma located in the isthmus or upper third is associated with Delphian lymph node metastasis. World J Surg.

[13] Iyer NG, Kumar A, Nixon IJ, Patel SG, Ganly I, Tuttle RM (2011). Incidence and significance of Delphian node metastasis in papillary thyroid cancer. Ann Surg.

[14] Kim WW, Yang SI, Kim JH, Choi YS, Park YH, Kwon SK (2012). Experience and analysis of Delphian lymph node metastasis in patients with papillary thyroid carcinoma. World J Surg Oncol..

[15] Lee YC, Shin SY, Kwon KH, Eun YG (2013). Incidence and clinical characteristics of prelaryngeal lymph node metastasis in papillary thyroid cancer. Eur Arch Otorhinolaryngol.

[16] Oh EM, Chung YS, Lee YD (2013). Clinical significance of Delphian lymph node metastasis in papillary thyroid carcinoma. World J Surg.

[17] Tan Z, Ge MH, Zheng CM, Wang QL, Nie XL, Jiang LH (2017). The significance of Delphian lymph node in papillary thyroid cancer. Asia Pac J Clin Oncol.

[18] Zheng G, Zhang H, Hao S, Liu C, Xu J, Ning J (2017). Patterns and clinical significance of cervical lymph node metastasis in papillary thyroid cancer patients with Delphian lymph node metastasis. Oncotarget..

[19] DeGroot LJ, Jameson JL (2006). Endocrinology.

[20] Russell RC, Williams NS, Bulstrode CJ (2004). Bailey and love’s short practice of surgery.

[21] Zheng GB, Hao SL, Liu XC, Ning JY, Wu GC, Wang D (2016). The clinical significance of Delphian lymph node metastasis in papillary thyroid cancer. Zhonghua Er Bi Yan Hou Tou Jing Wai Ke Za Zhi.

[22] Stang A (2010). Critical evaluation of the Newcastle-Ottawa scale for the assessment of the quality of nonrandomized studies in meta-analyses. Eur J Epidemiol.

[23] Higgins JP, Thompson SG (2002). Quantifying heterogeneity in a meta-analysis. Stat Med.

[24] Ferlito A, Shaha AR, Rinaldo A (2002). Prognostic value of Delphian lymph node metastasis from laryngeal and hypopharyngeal cancer. Acta Otolaryngol.

[25] Wierzbicka M, Leszczyńska M, Młodkowska A, Szyfter W, Bartochowska A (2012). The impact of prelaryngeal node metastases on early glottic cancer treatment results. Eur Arch Otorhinolaryngol.

[26] Haugen BR, Alexander EK, Bible KC, Doherty GM, Mandel SJ, Nikiforov YE (2016). 2015 American Thyroid Association management guidelines for adult patients with thyroid nodules and differentiated thyroid Cancer: the American Thyroid Association guidelines task force on thyroid nodules and differentiated thyroid Cancer. Thyroid..

[27] Lee SH, Lee SS, Jin SM, Kim JH, Rho YS (2008). Predictive factors for central compartment lymph node metastasis in thyroid papillary microcarcinoma. Laryngoscope..

[28] Roh JL, Park JW, Jeong J, Gong G, Cho KJ, Choi SH (2017). Extranodal extension of lymph node metastasis as a prognostic indicator of recurrence and survival in papillary thyroid carcinoma. J Surg Oncol.

[29] Liu FH, Kuo SF, Hsueh C, Chao TC, Lin JD (2015). Postoperative recurrence of papillary thyroid carcinoma with lymph node metastasis. J Surg Oncol.

[30] Ito Y, Miyauchi A, Kihara M, Kobayashi K, Miya A (2014). Prognostic values of clinical lymph node metastasis and macroscopic extrathyroid extension in papillary thyroid carcinoma. Endocr J.

