# Supplementary figures and images for: Clinical implications of Delphian lymph node metastasis in papillary thyroid carcinoma: a single-institution study, systemic review and meta-analysis
Source: J Otolaryngol Head Neck Surg. 2019 Aug 30;48:42. doi: 10.1186/s40463-019-0362-7 (PMC6716924; doi:10.1186/s40463-019-0362-7)

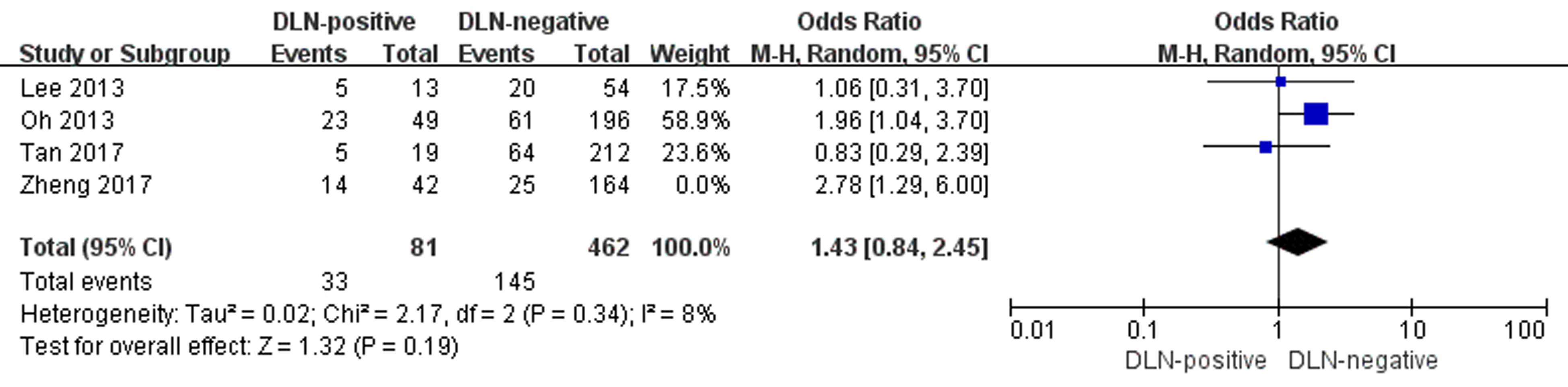

Supplement: Supplementary file 1 — Figure S1. The sensitivity analysis of bilaterality with and without Delphian lymph node metastasis. (PNG 771 kb) [file 40463_2019_362_MOESM1_ESM.png]

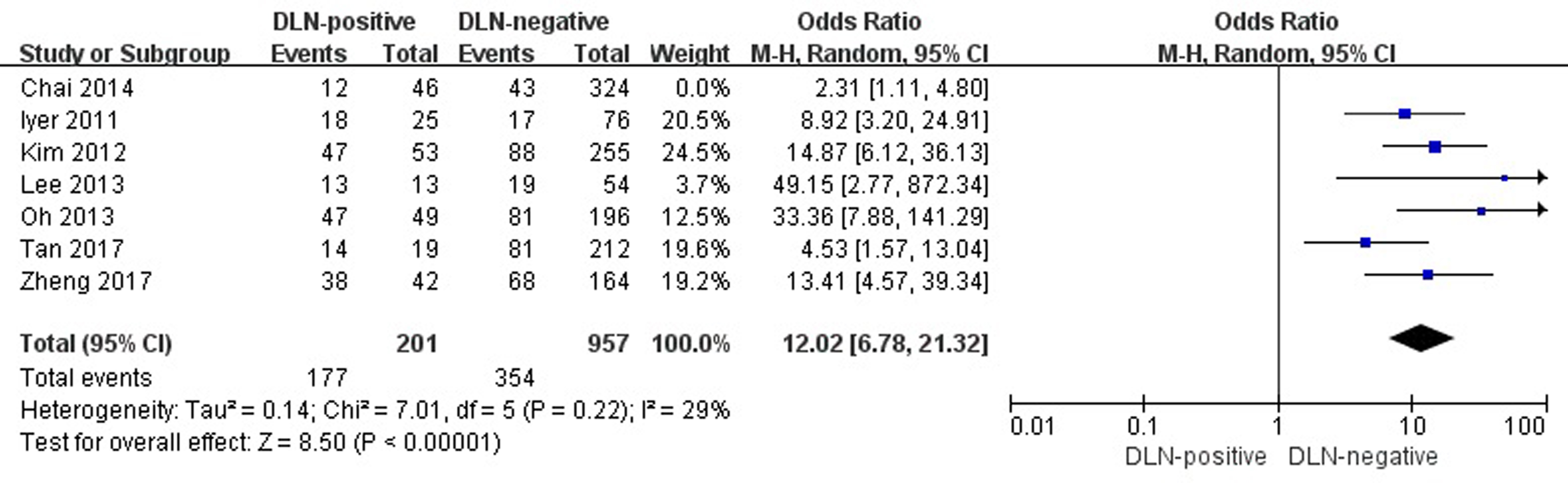

Supplement: Supplementary file 2 — Figure S2. The sensitivity analysis of further central lymph node metastasis with and without Delphian lymph node metastasis. (PNG 836 kb) [file 40463_2019_362_MOESM2_ESM.png]

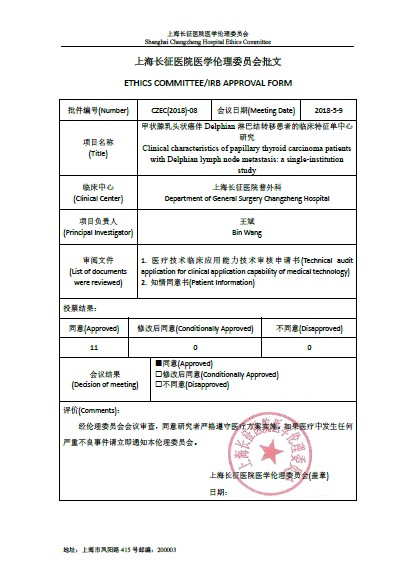

Supplement: Supplementary file 3 — Ethics approval from the local Clinical Ethics Committee. (JPG 59 kb) [file 40463_2019_362_MOESM3_ESM.jpg]
